# Protective Role of Alpha-Lipoic Acid Against Methotrexate-Induced Osteotoxicity: Mechanisms of Oxidative Stress Regulation and MAPK Pathway Inhibition

**DOI:** 10.3390/ph19050729

**Published:** 2026-05-05

**Authors:** Ahmet Can Haskan, Muhammed Said Altun, Osman Fatih Arpağ, Fariz Selimli, Soner Mete, Percin Pazarci, Halil Mahir Kaplan

**Affiliations:** 1 Department of Oral and Maxillofacial Surgery, Faculty of Dentistry, Hatay Mustafa Kemal University, 31001 Hatay, Turkey; ahmetcan.haskan@mku.edu.tr (A.C.H.); fariz.selimli@mku.edu.tr (F.S.); 2 Department of Periodontology, Faculty of Dentistry, Hatay Mustafa Kemal University, 31001 Hatay, Turkey; osmanfatiharpag@gmail.com; 3 Medical Promotion and Marketing Program, Department of Medical Services and Techniques, Medical Vocational Higher Services School, Nevsehir Hacibektas University, 50300 Nevşehir, Turkey; sonermete@nevsehir.edu.tr; 4 Department of Medical Biology, Faculty of Medicine, Cukurova University, 01330 Adana, Turkey; percinpazarci@gmail.com; 5 Department of Pharmacology, Faculty of Medicine, Cukurova University, 01330 Adana, Turkey; mkaplan@cu.edu.tr

**Keywords:** alpha-lipoic acid, osteotoxicity, methotrexate, oxidative stress, MAPK

## Abstract

**Background/Objectives**: Osteotoxicity is a severe complication of Methotrexate (MTX) chemotherapy, characterized by oxidative stress and disrupted bone remodeling. The primary objective of this study was to investigate the cytoprotective mechanisms of the antioxidant Alpha-Lipoic Acid (ALA) against MTX-induced osteotoxicity, specifically focusing on its modulation of oxidative stress, apoptosis, and Mitogen-Activated Protein Kinase (MAPK) signaling pathways. **Methods**: Murine osteocyte-like MLO-Y4 cells were cultured and exposed to a fixed dose of MTX (10^−5^ M), either alone or concurrently with ALA (50 μmol/L) for 48 h. Biochemical profiling was performed using specific enzyme-linked immunosorbent assays (ELISA) and colorimetric kits to evaluate pro- and anti-apoptotic proteins (Caspase-3, Bax, Bcl-2, Wee1, GRP78, GADD153, AIF), active MAPK components (p-JNK, p-ERK), and standard oxidative stress parameters (TAS, TOS, SOD, GPx). **Results**: MTX treatment induced significant cellular stress, evidenced by elevated Caspase-3, Bax, p-JNK, and p-ERK levels, alongside a critical reduction in Bcl-2 expression. MTX also markedly increased TOS while depleting TAS, SOD, and GPx levels. Conversely, co-treatment with ALA significantly mitigated these cytotoxic responses. ALA restored the Bax/Bcl-2 balance, effectively downregulated both p-JNK and p-ERK activation, and substantially reinforced the cellular antioxidant defense system by enhancing TAS, SOD, and GPx activities, although recovery to baseline control levels was partial. **Conclusions**: ALA exerts robust in vitro cytoprotective effects against MTX-induced osteotoxicity in MLO-Y4 cells by counteracting oxidative stress and inhibiting aberrant apoptotic and MAPK signaling. These findings establish a mechanistic baseline, underscoring the need for subsequent in vivo dose–response studies to validate ALA’s therapeutic potential in chemotherapy management.

## 1. Introduction

Osteotoxicity, commonly known as skeletal toxicity, refers to the adverse impacts that exogenous agents—termed osteotoxicants—exert on bone tissue throughout its development and structural maintenance [1,2]. Such compounds—whether encountered environmentally, consumed via a polluted diet, or entering the body through wounds—are capable of inducing severe skeletal malformations and compromising vital physiological mechanisms like bone mineralization, morphogenesis, and remodeling [3,4].

Methotrexate (MTX), a commonly used chemotherapeutic agent, has been associated with significant bone deficiencies. The osteotoxic nature of MTX is largely driven by its suppressive action on osteoblasts, the primary cells orchestrating bone formation. Consequently, the most prominent clinical outcomes of this damage are a significant decline in bone mineral density (BMD) and a greater propensity for skeletal fractures [5]. By hindering the proliferation and function of osteoblasts, MTX disrupts normal bone remodeling processes and induces a shift towards enhanced osteoclastogenesis, resulting in increased bone resorption [6]. The ensuing imbalance between bone formation and resorption lowers BMD, thereby weakening the skeletal structure and elevating fracture susceptibility. Given its widespread clinical application in both oncology and the management of chronic inflammatory diseases, long-term MTX exposure presents a significant hurdle for patient care. The literature extensively documents that MTX induces a severe imbalance in bone remodeling, primarily by generating excessive reactive oxygen species (ROS) that directly inhibit osteoblast differentiation while simultaneously promoting osteoclastogenesis [7]. Despite these well-documented effects on bone-forming and bone-resorbing cells, the specific damage inflicted upon the osteocyte network—the primary regulatory cells of the bone—remains underexplored. Osteocytes are the most abundant cells in bone tissue and function as the central orchestrators of bone remodeling, continuously regulating the activities of both osteoblasts and osteoclasts. Oxidative stress and apoptosis within the osteocyte population can severely disrupt the communication networks essential for maintaining skeletal homeostasis. Therefore, investigating the specific effects of MTX on osteocyte viability and the potential protective mechanisms of antioxidants like ALA is critical for a comprehensive understanding of MTX-induced osteotoxicity. Therefore, it is imperative to rigorously track the skeletal health of patients receiving MTX treatment and to adopt prophylactic measures aimed at preserving bone mineral density and overall structural integrity [8].

Recent research has illuminated the role of oxidative stress in mediating MTX’s osteotoxic effects, with excessive reactive oxygen species (ROS) being a central player [9]. Excessive ROS generation induces cellular apoptosis and activates inflammatory signaling networks. This oxidative burden, combined with abnormal levels of miRNAs and pro-inflammatory cytokines, ultimately stimulates osteoclastogenesis and severely hinders osteoblast development. This process is intricately linked to the activation of the Mitogen-Activated Protein Kinase (MAPK) signaling pathway, which responds to oxidative stress and may exacerbate bone damage [10].

In this context, alpha-lipoic acid (ALA) has emerged as a noteworthy antioxidant that may offer protective effects against oxidative stress-related harm [11,12]. ALA is known for its role in cellular energy metabolism and has garnered attention for its potential to mitigate the adverse effects of MTX on bone health. Through the effective neutralization of reactive oxygen species and the scavenging of free radicals, ALA mitigates the adverse effects of MTX. This robust antioxidant activity preserves osteoblast viability and functionality, thereby sustaining physiological bone turnover and synthesis. Moreover, ALA’s capacity to modulate inflammatory pathways and inhibit excessive osteoclast activity further contributes to its protective role in preserving bone integrity and preventing unnecessary bone loss.

While the general capacity of various antioxidants to mitigate MTX-induced toxicity has been previously reported, the precise intracellular signaling mechanisms mediating this protection specifically within osteocytes remain a critical knowledge gap [13]. Therefore, the primary objective of this study is to explicitly address this mechanistic gap. Specifically, the exact interplay between MTX-induced oxidative stress, the MAPK signaling cascade (JNK and ERK), and endoplasmic reticulum (ER) stress-mediated apoptosis requires thorough elucidation [14]. Therefore, this study aims to explicitly address this mechanistic gap by investigating how alpha-lipoic acid modulates not only the standard oxidative stress parameters, but specifically the MAPK pathway and a comprehensive panel of intracellular apoptotic mediators (including Wee1, a key kinase that regulates the G2/M cell cycle transition; GRP78 (Glucose-Regulated Protein 78), a master chaperone of the endoplasmic reticulum (ER) and a hallmark of the unfolded protein response (UPR); GADD153 (also known as CHOP), a pro-apoptotic transcription factor specifically induced by persistent ER stress; and AIF (Apoptosis-Inducing Factor), a mitochondrial protein that, upon release, triggers DNA fragmentation in a caspase-independent manner) in MTX-treated MLO-Y4 osteocyte-like cells. Understanding these specific targeted interactions is essential for developing effective strategies that safeguard bone integrity.

## 2. Results

**Figure 1:**
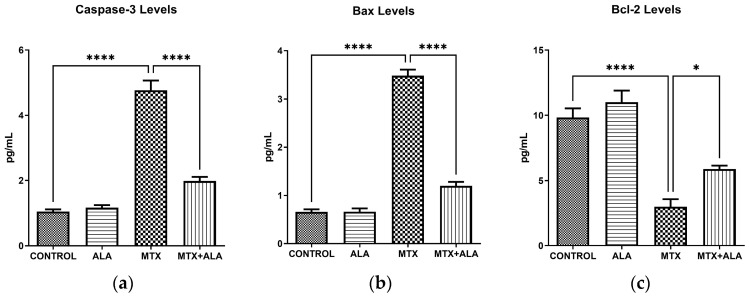
Impact of MTX and ALA treatments on key apoptotic mediators. Intracellular concentrations of (**a**) Cleaved Caspase-3, (**b**) Bax, and (**c**) Bcl-2 were quantified utilizing ELISA. Results are expressed as the mean ± SEM (*n* = 6 per group). One-way ANOVA coupled with Bonferroni’s post-hoc test was employed for statistical evaluations. (****: *p* < 0.0001, *: *p* < 0.05).

This investigation focused on defining the role of specific mediators central to programmed cell death. By measuring caspase-3 activity and corresponding protein expressions, we aimed to map their apoptotic contributions [15]. It is well-established that Bax and caspase-3 promote cell death, whereas Bcl-2 provides a vital survival signal [16]. Following MTX administration, we observed a profound induction of both caspase-3 (Figure 1a, *p* < 0.0001) and Bax (Figure 1b, *p* < 0.0001). This was accompanied by a sharp decline in Bcl-2 levels (Figure 1c, *p* < 0.0001), resulting in a pathological Bax/Bcl-2 balance that ultimately drives the cells toward apoptosis. Beyond these primary markers, MTX also triggered the upregulation of Wee1, GRP78, GADD153, and AIF (Table 1). Importantly, co-treatment with ALA effectively attenuated all of these adverse apoptotic responses.

It is important to note the overall extent of the recovery provided by the antioxidant treatment. Across all evaluated parameters—including apoptotic mediators, MAPK signaling components, and oxidative stress markers—the co-treatment with ALA (MTX+ALA group) yielded a highly significant improvement compared to the MTX-only group. However, statistical comparisons between the MTX+ALA group and the baseline Control group revealed that these values did not entirely return to normal baseline levels. This indicates that while the cytoprotective effect of ALA is robust and biologically significant, the recovery achieved at this specific dose is partial rather than absolute.

**Figure 2:**
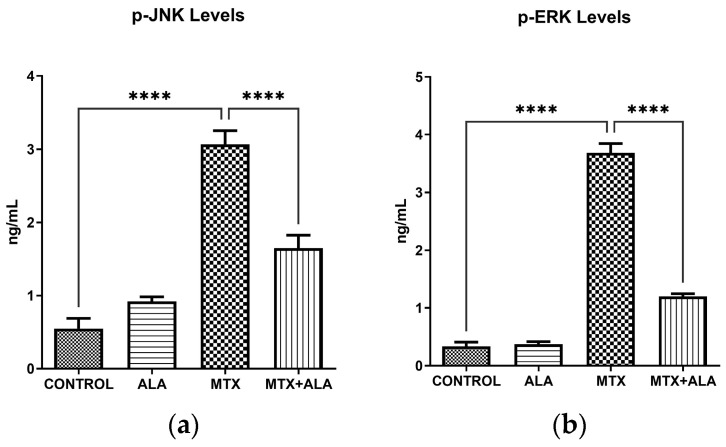
Effect of MTX and ALA on MAPK mediators. Intracellular concentrations of (**a**) p-JNK and (**b**) p-ERK were quantified utilizing ELISA. Results are expressed as the mean ± SEM (*n* = 6 per group). One-way ANOVA coupled with Bonferroni’s post-hoc test was employed for statistical evaluations. (****: *p* < 0.0001).

The MAPK signaling pathways, particularly JNK (c-Jun N-terminal kinase) and ERK (extracellular signal-regulated kinase), are central transducers of extracellular stimuli to the nucleus. p-JNK is typically recognized as a stress-activated protein kinase that responds to DNA damage and oxidative stress to promote apoptosis. In contrast, while p-ERK is often associated with pro-survival signals, its pathological hyperactivation—as observed in this study—is a documented response to severe chemical stress that can drive cell cycle arrest and reinforce the apoptotic cascade [17,18]. Our analysis revealed a profound elevation in the active forms of both JNK (p-JNK) and ERK (p-ERK) following MTX exposure when contrasted with the baseline control subjects. Importantly, concurrent treatment with ALA successfully suppressed this MTX-driven hyperactivation, significantly reducing the levels of both p-JNK (Figure 2a, *p* < 0.0001) and p-ERK (Figure 2b, *p* < 0.0001).

**Figure 3:**
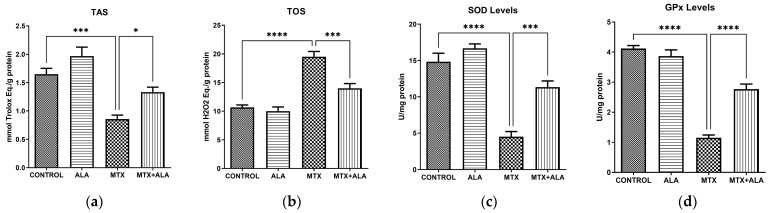
Effect of MTX and ALA on oxidative stress parameters. Intracellular concentrations of (**a**) TAS, (**b**) TOS, (**c**) SOD and (**d**) GPx were quantified utilizing ELISA. Results are expressed as the mean ± SEM (*n* = 6 per group). One-way ANOVA coupled with Bonferroni’s post-hoc test was employed for statistical evaluations. (****: *p* < 0.0001, ***: *p* < 0.001, *: *p* < 0.05).

Our investigation into oxidative stress dynamics demonstrated that MTX severely compromises the cellular redox balance. Specifically, MTX caused a sharp decline in Total Antioxidant Status (TAS) and a corresponding surge in Total Oxidant Status (TOS) compared to the untreated controls. The administration of ALA significantly counteracted this oxidative burden, boosting TAS (Figure 3a, *p* < 0.0001) and suppressing TOS (Figure 3b, *p* < 0.0001) relative to the MTX group. Similarly, the pivotal ROS-scavenging enzymes, superoxide dismutase (SOD) (Figure 3c, *p* < 0.0001) and glutathione peroxidase (GPx) (Figure 3d, *p* < 0.0001), exhibited drastically reduced activities following MTX exposure. Co-treatment with ALA successfully rescued these key endogenous defenses, resulting in markedly higher enzyme activities than those observed in the MTX-treated cells.

## 3. Discussion

This research explored how MTX administration impacts essential enzymes and proteins governing programmed cell death. Our results show that MTX strongly triggers apoptosis in target cells, as evidenced by a substantial surge in caspase-3 activity. This apoptotic drive is further reinforced by a simultaneous upregulation of the pro-apoptotic marker Bax. Importantly, we detected a severe downregulation of the crucial anti-apoptotic protein Bcl-2. This decline critically impairs the cellular defense mechanisms by shifting the Bax/Bcl-2 ratio toward a highly pro-apoptotic state, ultimately confirming that MTX exposure significantly accelerates cell death in these populations.

Current evidence emphasizes the dose-dependent benefits of ALA, showing that minimal concentrations enhance cell survival and defend against oxidative toxicity. This protective phenomenon is well-documented in various cellular environments, such as lung tumors, where ALA actively prevents apoptosis triggered by cytotoxic chemicals [19,20]. Mechanistically, ALA achieves this defense through its powerful ROS-scavenging capabilities [11]. It limits widespread oxidative damage by eliminating superoxide radicals and depleting intracellular ferrous iron, which together restrict the formation of harmful hydroxyl radicals. Despite these benefits, ALA acts as a double-edged sword; emerging studies show that administrating high doses can paradoxically stimulate apoptosis. This complex behavior means that therapeutic applications of ALA must be carefully titrated. Since the biological outcome shifts dramatically from cellular protection at low levels to active cytotoxicity at higher concentrations, future investigations must pinpoint the exact conditions that determine ALA’s ultimate effect.

Analyzing the significant accumulation of specific intracellular markers, such as Wee1, GRP78, GADD153, and AIF, reveals the diverse routes through which MTX inflicts cellular damage. Increased Wee1 levels halt cell cycle progression, preventing normal cellular proliferation. Concurrently, the induction of GRP78 and GADD153 highlights a severe endoplasmic reticulum stress response, and the surge in AIF confirms the involvement of alternative, caspase-independent apoptotic mechanisms [21,22,23]. The fact that ALA successfully mitigates these specific stress responses underscores its significant cytoprotective potential. Ultimately, ALA protects overall cellular viability within this in vitro system by re-establishing the equilibrium of these apoptotic mediators, reinforcing its value as a potent cellular protectant.

A broad spectrum of extracellular and intracellular triggers can initiate MAPK signaling networks [24]. Notably, reactive oxygen species (ROS) are profound stimulators of these pathways, despite the fact that the definitive pathways connecting ROS generation to MAPK activation are not yet completely mapped [25]. Within the cellular environment, ROS act as critical regulatory molecules [26]. Accumulating data highlight their capacity to function as secondary messengers, directly influencing major biological outcomes including cellular growth, specialization, and apoptosis. This dichotomous nature—serving as indispensable signaling conduits on one hand and inducing oxidative toxicity on the other—emphasizes their pivotal influence on cellular adaptation to external stressors [27].

Analyzing the MAPK cascades provides essential context for MTX-induced cytotoxicity. In cells exposed to MTX, we observed a striking upregulation of both p-JNK and p-ERK, clearly indicating the triggering of stress-responsive networks. The dual functionality of the ERK pathway warrants special attention. Although ERK signaling typically promotes cell growth and survival, its ultimate biological impact relies heavily on the strength and chronicity of the external insult [17]. When subjected to extreme oxidative and chemical stress—like MTX exposure—the persistent and abnormal hyperactivation of ERK paradoxically shifts the cell toward apoptosis and cycle arrest instead of survival [28]. Consequently, the high p-ERK expression noted in our MTX cohort represents a destructive, stress-driven phenomenon. When ALA was administered, the subsequent decline in p-ERK did not signify an impairment of normal survival functions; rather, it demonstrated the re-establishment of cellular equilibrium and the suppression of pathological apoptotic signals. ALA’s ability to concurrently block JNK and ERK hyperactivation firmly establishes its cytoprotective capacity. By normalizing these critical pathways, ALA simultaneously alleviates oxidative injury and programmed cell death, preserving cellular integrity during chemotherapy. Ultimately, targeting these dual mechanisms offers a promising therapeutic avenue to counteract the adverse effects of chemotherapeutic agents.

Analyzing oxidative stress markers provides a profound understanding of how the cellular landscape shifts during MTX administration [29]. The marked depletion of Total Antioxidant Status (TAS) coupled with the surge in Total Oxidant Status (TOS) demonstrates that MTX provokes a severe oxidative burden, which ultimately drives cellular injury and programmed death. Such redox disequilibrium is widely recognized as a primary driver of pharmacological toxicity. Moreover, the steep decline in superoxide dismutase (SOD) and glutathione peroxidase (GPx) functions underscores the intense oxidative pressure placed on the MTX-treated cells. Because these specific enzymes are essential for neutralizing reactive oxygen species, their suppression clearly indicates a collapse of the cell’s intrinsic antioxidant defense mechanisms.

ALA’s efficacy in restoring TAS and reducing TOS levels underscores its role as a potent antioxidant. The observed increase in SOD and GPx activities in the ALA-treated group further supports the hypothesis that ALA enhances cellular antioxidant defenses, thereby reducing oxidativedamage and preventing apoptosis. This enhancement of the cellular antioxidant defense system suggests that ALA actively contributes to the preservation of cellular integrity in the face of oxidative challenges posed by MTX.

While our assessment of Total Oxidant Status (TOS) provides a robust quantification of the cumulative oxidative burden and implies elevated ROS production, a limitation of the current experimental design is the absence of real-time, dynamic ROS generation tracking in live cells. Future investigations utilizing direct intracellular fluorescent ROS probes or flow cytometry would provide valuable functional endpoints to further elucidate the exact kinetics of MTX-induced ROS generation and the specific temporal scavenging efficiency of ALA.

Overall, this study demonstrates that while MTX induces significant apoptotic signaling through various pathways, including the upregulation of pro-apoptotic proteins and the activation of MAPK pathways, ALA can effectively counteract these effects. By restoring the balance of pro-apoptotic and anti-apoptotic factors, inhibiting the MAPK pathway activation, and reinforcing the cellular antioxidant capacity, ALA effectively mitigates these specific cytotoxic responses in cultured osteocyte-like cells.

While our study provides robust biochemical evidence demonstrating the protective effects of ALA against MTX-induced osteotoxicity, several methodological limitations must be acknowledged. Our protein quantification relied primarily on ELISA without orthogonal validation via Western blot or RT-PCR. Furthermore, the study lacks real-time tracking of intracellular ROS generation and phenotypic cell viability assays, such as MTT. Future investigations utilizing direct fluorescent ROS probes or flow cytometry are essential to elucidate the exact kinetics of MTX-induced oxidative stress and the temporal scavenging efficiency of ALA. Additionally, as we utilized a fixed-dose in vitro model, these mechanistic findings cannot be directly extrapolated to clinical settings without subsequent in vivo research using animal models and dose–response matrices.

Moreover, while a strong association was observed between ALA treatment and the downregulation of MAPK and ER stress pathways, the absence of specific pharmacological inhibitors or genetic knockdown models limits our ability to establish a definitive causal hierarchy. Future studies employing targeted inhibitors are strictly necessary to fully map the mechanistic sequence of this signaling cascade. Despite these limitations, our findings establish a critical baseline for the cytoprotective potential of ALA. By identifying the conditions that optimize ALA’s antioxidant properties while minimizing potential cytotoxicity, future clinical research can better harness this agent to preserve bone health in patients undergoing chemotherapy.

## 4. Materials and Methods

### 4.1. Chemicals

The culture media used in this research were obtained from GIBCO BRL (Grand Island, NY, USA), while calf serum was sourced from HyClone Laboratories, Inc. (Logan, UT, USA). Rat tail collagen type I was purchased from Becton Dickinson Laboratories (Bedford, MA, USA). Reagents such as Radioimmunoprecipitation Assay (RIPA) buffer, fetal bovine serum, bovine serum albumin, Phosphate-Buffered Saline (PBS), NaCl, TritonX-100, Ethylene Glycol Tetraacetic Acid (EGTA), dithiothreitol, NaF, Tris–HCl, and sodium vanadate were acquired from Sigma-Aldrich (St. Louis, MO, USA). ELISA kits for the analysis of bax (cat#: 201-02-1544), bcl-2 (cat#: 201-02-0477), Wee1 (cat: abx595628), AIF (cat#: 201-02-0138), GADD153 (cat#: 201-12-5342), and GRP78 (cat#: SRB-T-87954) were provided by Shanghai Sunred Biological Technology Co., Ltd. (Shanghai, China). Active ERK (p-ERK) (cat#: MBS3805597) and JNK (p-JNK) (cat#: MBS733822) detection kits were sourced from My BioSource, Inc. (San Diego, CA, USA). SOD (cat#: K335-100) and GPX (cat#: K762-100) assay kits originated from BioVision (Milpitas, CA, USA), while assays for Total Antioxidant Status (TAS) and Total Oxidant Status (TOS) were obtained from Rel Assay Diagnostics Inc. (Gaziantep, Turkey). The Bradford dye reagent was supplied by Bio-Rad Laboratories, Inc. (Hercules, CA, USA).

### 4.2. Cell Culture

The murine osteocyte-like cell line MLO-Y4 (cat#: EKC002) was acquired from Kerafast, Inc. (Newark, CA, USA). MLO-Y4 cells utilized between passages 15–20 were cultured in Dulbecco’s Modified Eagle Medium (DMEM) supplemented with 10% fetal bovine serum. To maintain their dendritic phenotype, the cells were cultured on plates coated with rat tail collagen type I, strictly following the methodology established by Kato et al. [30]. For the experiments, MLO-Y4 cells were seeded into six-well plates at a density of 1 × 10^5^ cells/well and allowed to attach overnight. The cells were exposed to MTX at a concentration of 10^−5^ M, a dose established in prior literature to effectively induce oxidative damage and apoptosis without immediate necrosis. MTX was administered both individually and concurrently with ALA at 50 μmol/L for a duration of 48 h. The 50 μmol/L concentration of ALA was specifically selected as it represents an optimal sub-cytotoxic dose that maximizes antioxidant cytoprotection while avoiding the pro-apoptotic effects associated with higher concentrations [31,32]. This fixed-dose approach was employed to establish a foundational mechanistic baseline for the current in vitro study. The designated Control group was cultured under strictly identical conditions and treated with an equivalent volume of the vehicle solvent (0.1% DMSO) to exclude any potential solvent-induced cytotoxicity or oxidative stress. After treatment, cells were homogenized for ELISA analysis.

### 4.3. Cell Homogenization

Following a 48-h incubation period, cells from the experimental groups (ALA, MTX, and ALA+MTX) cultivated in six-well plates were harvested into 15 mL conical tubes. The samples underwent initial centrifugation at 2000 rpm for 10 min at 4 °C to pellet the cells, after which the culture media supernatant was aspirated. The resulting cell pellets were washed by resuspension in 5 mL of cold PBS, followed by a second centrifugation step under identical conditions (2000 rpm, 10 min, 4 °C) and subsequent removal of the PBS wash. For protein extraction, the washed pellets were lysed using a mixture containing 250 μL of RIPA buffer supplemented with 2.5 μL of PMSF (200 mM), 2.5 μL of sodium vanadate (100 mM), and 2.5 μL of protease inhibitor cocktail. The cell suspensions were subsequently disrupted on ice using an ultrasonic homogenizer. Finally, the lysates were cleared by centrifugation at 10,000 rpm for 10 min, allowing for the collection of the protein-rich supernatants while the cellular debris pellets were discarded.

### 4.4. Total Protein Determination

To determine the total protein content within the cellular homogenates, a standard Bradford assay was employed. Bovine serum albumin (BSA) served as the reference standard across a concentration range of 1–100 μg/μL. The final protein concentrations of the samples (expressed in μg/μL) were derived by interpolating the data against a standard curve, which was constructed using GraphPad Prism software (version 9.0).

### 4.5. ELISA (Enzyme-Linked Immunosorbent Assay) Tests

Intracellular levels of active signaling kinases (p-ERK and p-JNK), alongside core apoptotic regulators (caspase-3, Bax, Bcl-2, Wee1, GADD153, and AIF), were systematically measured using specific ELISA methodologies. Concurrently, redox balance parameters, including TAS, TOS, SOD, and GPx, were quantified through dedicated colorimetric techniques. All experimental steps for both the immunosorbent and colorimetric evaluations were carried out exactly as outlined in the manufacturers’ official instruction manuals.

### 4.6. Statistical Analysis

The experimental data are reported as the mean ± S.E.M. For all independent experiments, ‘n’ refers to the number of individual cell culture flasks per treatment condition. Statistical evaluations between multiple groups were carried out using analysis of variance (ANOVA), supplemented by Bonferroni’s multiple comparison test. Differences were deemed statistically significant when the *p*-value fell below the 0.05 level. All the relevant data is given in Supplementary Material Data S1.

## 5. Conclusions

In conclusion, this study demonstrates that while MTX induces significant apoptotic signaling in MLO-Y4 osteocyte-like cells through oxidative stress and the activation of MAPK pathways, ALA effectively mitigates these cytotoxic responses. By restoring the balance of pro-apoptotic and anti-apoptotic factors and reinforcing the cellular antioxidant defense system, ALA helps preserve cellular integrity in vitro. These findings provide a promising mechanistic basis for utilizing ALA as a protective agent against MTX-induced osteotoxicity, highlighting the need for further clinical investigation to translate these results into improved management strategies for bone health during cancer treatment.

## Figures and Tables

**Table 1:** Modulatory effects of MTX and ALA exposure on the expression profiles of apoptotic proteins (*n* = 6).

	Control	ALA	MTX	MTX+ALA
wee 1	0.28 ± 0.02 pg/mL	0.27 ± 0.02 pg/mL	1.03 ± 0.05 pg/mL *	0.66 ± 0.03 pg/mL #
AIF	0.87 ± 0.04 pg/mL	0.86 ± 0.03 pg/mL	1.77 ± 0.05 pg/mL *	1.1 ± 0.05 pg/mL #
gadd153	0.29 ± 0.03 pg/mL	0.30 ± 0.05 pg/mL	1.11 ± 0.04 pg/mL *	0.52 ± 0.03 pg/mL #
grp78	0.36 ± 0.05 pg/mL	0.33 ± 0.06 pg/mL	1.89 ± 0.07 pg/mL *	1.02 ± 0.04 pg/mL #

The presented results represent the mean ± SE. For multiple statistical comparisons, a one-way ANOVA incorporating a Bonferroni correction was applied. Significance levels are defined as follows: *: *p* < 0.0001 when comparing MTX to the Control, and #: *p* < 0.0001 when comparing the combined MTX+ALA treatment to the MTX group.

## Data Availability

The original contributions presented in this study are included in the article/Supplementary Material. Further inquiries can be directed to the corresponding author.

## Supplementary Materials

The following supporting information can be downloaded at: https://www.mdpi.com/article/10.3390/ph19050729/s1. Data S1: Data for Figure 1, Figure 2 and Figure 3.
